# Case of Chronic Inflammation of the Stomach

**Published:** 1873-10

**Authors:** A. Fisher, James N. Hyde, I. N. Danforth


ARTICLE II.—*Case of Chronic Inflammation of the Stomach.* From the Transactions of the Chicago Society of Physicians and Surgeons; Meeting of June 14, 1873. By Drs. A. FISHER, JAMES N. HYDE, and I. N. DANFORTH.

Dr. A. Fisher gave the following details of a case of chronic inflammation of the stomach:

I have been acquainted with Mrs. I——, for the past sixteen years, and during that time have been her only physician. She was 49 years old, of a nervo-bilious temperament, and was always in delicate health, though never dangerously ill. She suffered from occasional attacks of intermittent fever, and generally had a red tongue, irritable stomach and dyspeptic symptoms, though she was able to discharge her household duties, and called herself well.

The following note, penned by her husband, describes her later condition:

"In the summer and fall of 1872, she complained frequently of a 'fullness' after eating, or very moderate drinking, and remarked several times, laughingly, 'if it were not absurd, I should say my throat was filling up.' During January and February of 1873, this feeling increased, and, towards the latter part of February, she began to lose her usual capacity, though not her desire, for food. Believing herself to be suffering from an aggravated form of dyspepsia, she tried very many remedies without success. In March, she gradually commenced the use of gruels, etc., as the only dietetic articles she could retain on her stomach, and was soon visited by that physical weakness which results from a low diet. All through March and early in April, she repeatedly remarked, 'I must get up, for I feel that if I should keep my bed for one day, I should not soon want to get up again.' On the 9th of April, when we moved, she was extremely weak and emaciated, and, on the 12th, was startled by your asking if she had any hereditary tendency to cancer in her family. On the 20th I described her condition to a friend as follows: Parched mouth, with coppery taste and canker sores—copious discharge of frothy phlegm—great thirst—burning pain in the chest—distress always after eating, sometimes after drinking, located under the breast-bone, and between the breasts—tendency to vomit food directly after eating, and, when the latter was retained, free vomiting of frothy matter—regular pulse and constipated bowels. On the night of June 6th, she commenced to discharge a fetid yellowish matter at intervals of about six hours. At ten o'clock A. M. of the 9th, this discharge ceased, but recommenced at seven o'clock the next morning, and was very copious, but not nearly so fetid as before. Her average pulse during the week that preceded her death, was 112—up to June 9th, the highest noticed was 122. From the 4th to the 10th of June, she had no stools, but frequent copious and painless urination. She had no *feeling* of fever, though numerous signs of its presence were not wanting. On the 10th of June, morphia was administered at eight o'clock A. M., and six P. M., at her urgent request; she was then failing fast, and died quietly at four o'clock A. M., June 11th."

Mrs. I—— called on me for a prescription last October, (I had not prescribed for her previous to that time for nearly one year), and said she thought her liver was affected. I made a careful examination, and then informed her that her stomach was the source of her trouble, and advised her to regulate her diet. I ordered for her bismuth and pepsine, which she thought for a time gave her relief. I prescribed for her two or three times in October, once in December, and three or four times in March, using those remedies which were indicated to allay the irritability of the stomach and to assist the digestion—this without any permanent benefit, the patient growing weaker and becoming more emaciated.

I was first called to see her in her last illness, on the 13th of April. I found her very much emaciated, with sallow skin, dry, red and smooth tongue, pulse 70 in the minute, with tenderness in the region of the stomach, and an irregular indurated mass, evident to the touch somewhat to the left of the pit of the stomach. She complained of a burning pain under the middle of the sternum, aggravated by the ingestion of food, which she did not believe entered the stomach. She took no solid food, and but a few spoonfuls of liquid nourishment at any one time, this amount generally producing vomiting of a glairy, frothy mucus, unmixed with either blood or pus. Her bowels were constipated, but moved daily by enemata.

I ordered eight grains of the subnitrate of bismuth every six hours, with sinapisms over the stomach, and a diet of beef-tea and mucilaginous drinks. In four days I saw her again; there was little less vomiting, and she was otherwise in no better condition. I then prescribed a pill of one-fourth of a grain of nitrate of silver, and two grains of the English extract of cicuta, every six hours, continuing at the same time the use of the bismuth—this with apparent benefit for one week. The "burning heat" was not so severe, though she felt that nothing remained in her stomach and had occasional attacks of vomiting.

On the 25th of April, Dr. N. S. Davis saw her with me in consultation. We agreed that there was thickening and contraction of the stomach, and, from the feel of the noduled induration, already described, as well as from the consideration of the history of the case, we were led to fear cancer of the stomach, but did not express a positive opinion on the subject.

Dr. Davis advised the continuation of the nitrate of silver and cicuta, and the substitution for the bismuth of one-fourth of a grain of carbolic acid, with four drops of the tincture of belladonna in syrup every six hours. This course was followed for one or two days, when either the carbolic acid or the belladonna irritated the stomach, and both were suspended; the pill being administered alone for about one week longer. At the expiration of that time, all medication was discontinued, as she could not retain even a teaspoonful of beef-tea in the stomach. Her mouth and lips were kept constantly moistened by water, and a teaspoonful of it given when called for.

Her sufferings were very great and grew constantly worse throughout her illness, and as a cure seemed impossible, I advised, at the last, hypodermic injections of morphia sufficiently often to relieve her of pain. They had a happy effect, and she continued to take them, increasing the dose according to the necessities of her case for about three weeks, until she died, June 11, 1873.

The autopsy was made by Dr. James N. Hyde, June 12, 1873.

There was great emaciation and cadaveric rigidity. The brain was not examined. The heart, lungs, liver, spleen, kidneys, pancreas, uterus and ovaries, were found to be in a condition entirely normal. The stomach was greatly contracted, especially at a point about one inch distant from the pyloric extremity, its walls were hypertrophied, its mucous membrane congested, thickened and partially eroded. There was no post-mortem digestion of its coats. A small cul-de-sac, on the left side and in the greater curvature, seemed to exhibit fewer traces of inflammatory products than any other portion, and was partially filled with a serous liquid. The œsophagus was not implicated in the diseased portions. The omentum was studded with small granules of the size of split peas, and, in the interstices of the latter, was moderately injected, but not greatly altered. The duodenum seemed quite free from any thickening of its coats, but, to a certain extent, participated in the condition which was evident in all other portions of the intestinal tract—congestion and traces of a low form of inflammatory action being everywhere present. The bladder was moderately distended with limpid urine.

The stomach was sent to Dr. I. N. Danforth for microscopical examination.

PATHOLOGY.

The walls of the stomach were thickened to an extreme degree; the organ was very much contracted, in fact, it was gathered up into a hard, irregular, "puckered" mass of folds and ridges, and was doubtless permanently retained in this condition. The mucous membrane was excessively hypertrophied, rough and brawny; it was successively elevated and depressed, as a consequence of the general contraction of the stomach, and these elevations and depressions, or "hills and valleys," (Rindfleisch), remained after the stomach was cut open and put upon the stretch.

MICROSCOPY.

I have examined several thin and carefully prepared transverse sections, extending through the entire thickness of the stomach, and find one uniform condition applying to them all.

*First.*—The connective tissue is enormously increased, not only the submucous layer and that beneath the muscular tissue, but also that delicate inter-connective tela which is associated with the gastric follicles. This new tissue is of course the product of an inflammatory proliferation of the connective tissue-cells; these cells, being stimulated by inflammatory action to an abnormally rapid growth, must necessarily produce tissue which is abnormal in *type* as well as in quantity, in accordance with the general law governing inflammatory proliferations. Indeed, this new tissue so nearly resembles ordinary cicatrix tissue, both in appearance and behavior, that we may regard them as practically identical.

*Second.*—The gastric follicles are all but destroyed; they have been slowly choked and squeezed out of *functional* existence by the progressive contraction of the newly formed connective tissue. In none of the sections examined, were the gastric follicles in anything like the natural size or condition, and generally they were so pinched and distorted as to have almost lost their identity. Hence, the inability to retain or digest food. But the same contraction which destroyed the gastric follicles, also involved the nerves of the stomach; they were subjected to exactly the same mechanical violence, and of course they uttered their protest in the only language known to them. Hence, the extreme pain of which the patient complained, and the resulting inference that the case was possibly one of cancer of the stomach.

We have, then, this sequence of events:

1st.—The development of inflammatory action, which was from the first of low "grade," and therefore all the less likely to attract sufficient attention, and cause sufficient discomfort to lead the patient to call a physician, or seek relief from a trouble which, in its incipiency, was probably regarded as hardly worth noticing.

2nd.—The proliferation of connective tissue-cells, producing a superabundance of tissue of low type, which so nearly resembled scar tissue that it behaved *like* scar tissue. Hence, the extreme and permanent contraction of the stomach. It is noticeable that the tissue primarily involved, namely, the connective tissue; and the pathological condition produced as a consequence thereof, namely, contraction, are precisely analogous to the events and consequences which we meet with in cirrhosis of the liver, and the small contracted kidney of one of the forms of Bright's disease.

